# Efficacy and safety of proprotein convertase subtilisin/kexin type 9 monoclonal antibody in adults with familial hypercholesterolemia

**DOI:** 10.18632/oncotarget.10762

**Published:** 2016-07-21

**Authors:** Bin Li, Pan-Pan Hao, Yong Zhang, Rui-Hong Yin, Qing-Zan Kong, Xiao-Jun Cai, Zhuo Zhao, Jian-Ni Qi, Ying Li, Jie Xiao, Fu Wang, Wei Yi, Xiao-Ping Ji, Guo-Hai Su

**Affiliations:** ^1^ Department of Cardiology, Jinan Central Hospital affiliated to Shandong University, Jinan, Shandong, China; ^2^ Key Laboratory of Cardiovascular Remodeling and Function Research, Chinese Ministry of Education and Chinese Ministry of Health, Department of Cardiology, Shandong University Qilu Hospital, Jinan, Shandong, China; ^3^ Department of Neurology, Jinan Central Hospital affiliated to Shandong University, Jinan, Shandong, China; ^4^ Department of Gastroenterology, First Peoples Hospital of Jinan, Jinan, Shandong, China; ^5^ Central Laboratory, Shandong Provincial Hospital affiliated to Shandong University, Jinan, Shandong, China; ^6^ Central Laboratory, Jinan Central Hospital affiliated to Shandong University, Jinan, Shandong, China; ^7^ Engineering Training Center, Shandong University, Jinan, Shandong, China

**Keywords:** efficacy, safety, proprotein convertase subtilisin/kexin type 9 monoclonal antibody, familial hypercholesterolemia

## Abstract

Proprotein convertase-subtilisin/kexin type 9 (PCSK9) monoclonal antibody is a new therapy to reduce low-density lipoprotein cholesterol (LDL-C) level in patients with familial hypercholesterolemia (FH). This pooled analysis aimed to estimate the efficacy and safety of PCSK9 antibody therapy in FH. Reports of randomized controlled trials (RCTs) comparing PCSK9 antibody to placebo were retrieved by a search of MEDLINE via PubMed, EMBASE, the Cochrane Library databases, ClinicalTrials.gov and Clinical Trial Results (up to November 30, 2015) with no language restriction. Data were abstracted by a standardized protocol. We found eight RCTs (1,879 patients with FH) for the pooled analysis. As compared with placebo, PCSK9 antibody therapy remarkably reduced LDL-C level (mean reduction: -48.54 %, 95 % CI: -53.19 to -43.88), total cholesterol (mean reduction: -31.08%, 95 % CI: -35.20 to -26.95), lipoprotein (a) (mean reduction: -20.44%, 95 % CI: -25.21 to -15.66), and apolipoprotein B (mean reduction: -36.32%, 95 % CI: -40.75 to -31.90) and elevated the level of high-density lipoprotein cholesterol (mean change: 6.29 %, 95 % CI: 5.12 to 7.46) and apolipoprotein A1(mean change: 4.86%, 95 % CI: 3.77 to 5.95). Therapy with and without PCSK9 antibodies did not differ in rate of adverse events (pooled rate: 50.86 % vs. 48.63%; RR: 1.03; 95 % CI: 0.92 to 1.15; *P* = 0.64; heterogeneity *P* = 0.13; *I*^2^= 40%) or serious adverse events (pooled rate: 7.14% vs. 6.74%; RR: 1.05; 95 % CI: 0.70 to 1.58; *P* = 0.80; heterogeneity *P* = 0.69; *I*^2^= 0%). PCSK9 antibody may be an effective and safe treatment for FH.

## INTRODUCTION

Familial hypercholesterolemia (FH) is a genetic disease involved in lipid metabolism caused by mutations in low-density lipoprotein receptor (LDLR), apolipoprotein B (ApoB) and proprotein convertase subtilisin/kexin type 9 (PCSK9) [1]. FH is clinically classified as heterozygous familial hypercholesterolemia (HeFH) and homozygous familial hypercholesterolemia (HoFH) [2]. The characteristics of patients with FH are elevated plasma level of low-density lipoprotein cholesterol (LDL-C) and increased risk of premature coronary heart disease [3, 4]. Statins are the first-line drugs for treatment of FH [5], but the guidelines recommending LDL-C goals are not achieved despite high-intensity statin therapy [6]. Combined treatment with high-strength statins and ezetimibe or other drugs may help lower LDL-C levels [7, 8], but achieving the treatment targets is difficult [3, 9–11]. As well, some patients fail to adhere to statins treatment because of its side effects [12].

PCSK9 is a kind of serine protease that is synthesized and secreted by the liver; it is expressed in the liver, small intestine, kidney and nervous system [13, 14]. PCSK9 binds to LDLR for LDLR degradation in lysosomes, which eventually elevates the plasma level of LDL-C [15, 16]. PCSK9 is connected to dyslipidemia, especially LDL-C metabolism [17], and is closely related to risk of coronary heart disease.

The FH phenotype is caused by gain-of-function mutations in PCSK9 [3, 15]. Inhibiting PCSK9 has led to potential therapeutic agents for FH [15, 18–20]. The use of PCSK9 monoclonal antibodies can reduce circulating LDL-C level in patients with FH and could be synergistic with statins [21].

The efficiency and safety of PCSK9 inhibitor therapy for hypercholesterolemia has been evaluated [22–24], but a pooled analysis of the therapy for FH is lacking. In addition, the efficacy outcomes for lipids in FH are inconsistent. Thus, we conducted a pooled analysis of randomized controlled trials (RCTs) to systemically evaluate the efficiency and safety of PCSK9 antibody therapy for FH.

## RESULTS

### Study selection and patient characteristics

**Figure 1:**
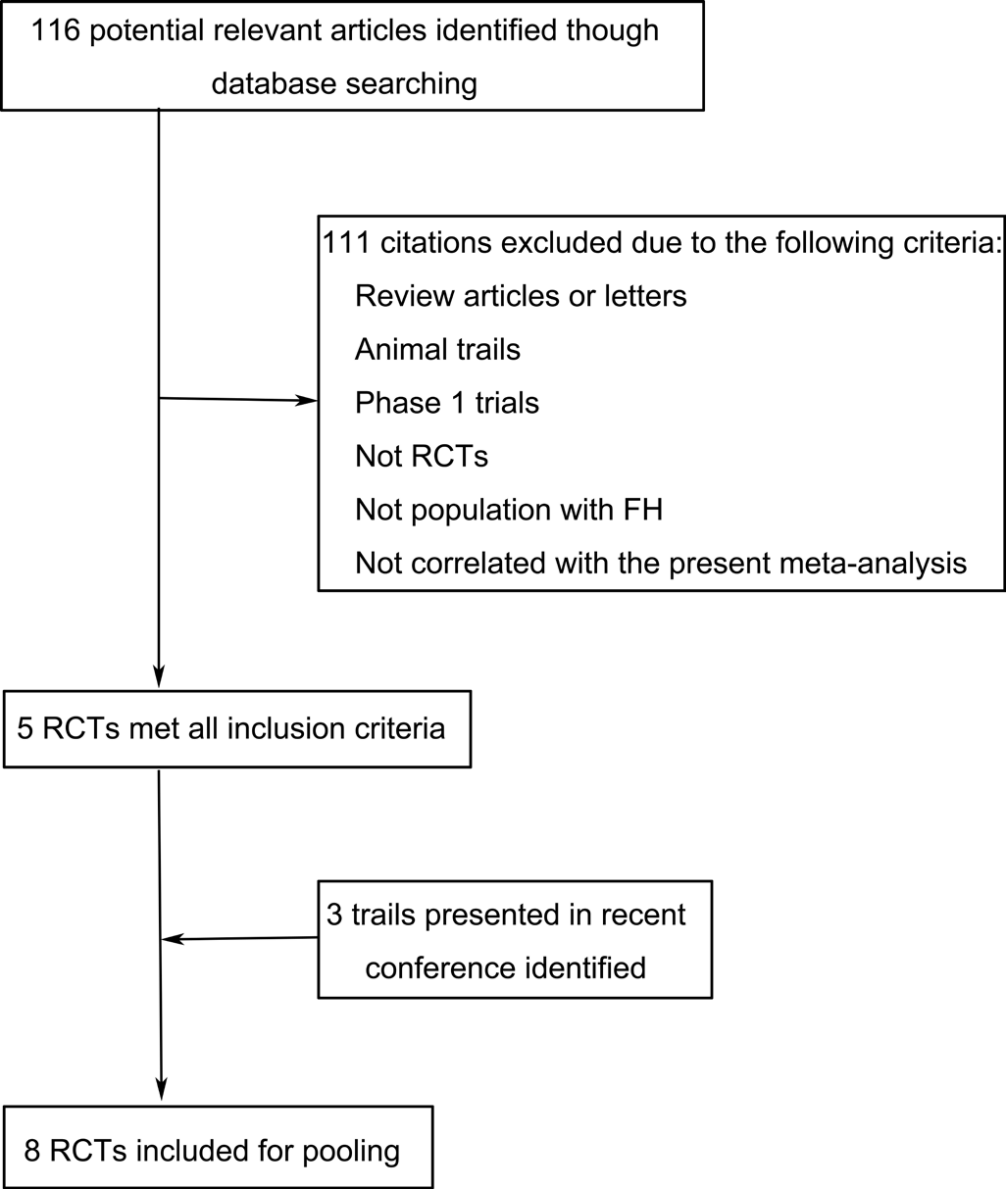
Flow chart for study selection RCT, randomized controlled trial; FH, familial hypercholesterolemia.

Our search retrieved 116 related studies in total; 111 were excluded because they were review articles, letters, animal trials, phase 1 trials, not RCTs, not population with FH, or were not correlated with the present pooled analysis. We included unpublished reports for three clinical trials (ODYSSEY FHI, ODYSSEY FHII and ODYSSEY HIGH FH) (Figure 1). Our final sample included reports for eight studies including 1,879 patients with FH. All eight studies were of good quality (Jadad score≥3).

Characteristics of the eight studies are in the Table 1. One study was of HoFH patients and seven were of HeFH patients. Two reports were of phase 2 trials and six were of phase 3 trials; Alirocumab was subcutaneously injected as PCSK9 antibody in five studies and evolocumab in three others; Four trials were 12 weeks and four were > 12 weeks long.

**Table 1:** Baseline characteristics of clinical trials

Study	Journal, Year	Phase	Patients, n	Mean age (y)	Women, n (%)	Duration (w)	Investigational drug and dose	Control	Population	LLT background
RUTHERFORD	Circulation, 2012	2	167	50 (13)	79 (47)	12	Evolocumab 350 mg Q4W and 420 mg Q4W	Placebo	HeFH	Statin ± ezetimibe
TESLA Part B	Lancet, 2014	3	49	31 (13)	24 (49)	12	Evolocumab 420 mg Q4W	Placebo	HoFH	Statin ± ezetimibe
RUTHERFORD-2	Lancet, 2014	3	329	51 (14)	139 (42)	12	Evolocumab 140 mg Q2W and 420 mg Q4W	Placebo	HeFH	Statin ± ezetimibe
Stein et al.	Lancet, 2012	2	77	53 (10)	30 (39)	12	Alirocumab 150, 200, or 300 mg Q4W and 150 mg Q2W	Placebo	HeFH	Statin ± ezetimibe
ODYSSEY FH I	ESC Congress 2014	3	486	52 (12)	212 (55)	24	Alirocumab 75 mg with potential up-titration to 150 mg Q2W	Placebo	HeFH	Statin ± other LLT
ODYSSEY FH II	ESC Congress 2014	3	249	53 (13)	118 (47)	24	Alirocumab 75 mg with potential up-titration to 150 mg Q2W	Placebo	HeFH	Statin ± other LLT
ODYSSEY HIGH FH	AHA Scientific Sessions 2014	3	107	52 (11)	50 (47)	24	Alirocumab 150 mg Q2W	Placebo	HeFH	Statin ± other LLT
ODYSSEY LONG TERM	NEJM, 2015	3	2341	61 (10)	884 (38)	24	Alirocumab 150 mg Q2W	Placebo	HeFH + HC	Statin ± other LLT

Data are mean (SD), number (%); Q2W, every 2 weeks; Q4W, every 4 weeks; HeFH, heterozygous familial hypercholesterolemia; HoFH, homozygous familial hypercholesterolemia; HC, hypercholesterolemia; LLT, lipid-lowering therapy.

### Clinical end points

#### Efficacy outcomes

We used all eight reports for the analysis of LDL-C with a random-effects model because of significant heterogeneity (*P* < 0.00001, *I*^2^ = 100%). Level of LDL-C were reduced almost 50% with than without PCSK9 antibody treatment (mean reduction: −48.54%, 95% confidence interval [CI]: −53.19 to −43.88) (Table 2). On subgroup analysis, LDL-C level was reduced more in patients with HeFH than HoFH (mean reduction: −51.03%, 95% CI: −55.59 to −46.48 *vs*. -31.00%, 95 %CI: -33.96 to −28.04). Heterogeneity tests for subgroups showed a striking difference between HeFH and HoFH groups *(P* < 0.00001), so the heterogeneity was caused in part by the different populations. However, analyses by type of PCSK9 antibody or duration of treatment did not reveal heterogeneity (Table 3).

**Table 2:** Pooled-analysis results of the percentage change in level of serum lipid and the incidence of adverse events

Outcomes	Patients, n	WMD/RR (95% CI)	*P* value	*I*^2^, %	Heterogeneity *P* value
LDL-C	1875	−48.54 %[-53.19, -43.88]	*P* < 0.00001	100%	*P* < 0.00001
HDL-C	1460	6.29 %[5.12, 7.46]	*P* < 0.00001	97%	*P* < 0.00001
TC	1082	−31.08%[-35.20, -26.95]	*P* < 0.00001	99%	*P* < 0.00001
Lp(a)	1383	−20.44%[-25.21, -15.66]	*P* < 0.00001	100%	*P* < 0.00001
ApoA1	1392	4.86%[3.77, 5.95]	*P* < 0.00001	97%	*P* < 0.00001
ApoB	1438	−36.32%[-40.75, -31.90]	*P* < 0.00001	100%	*P* < 0.00001
TG	1383	−7.92%[-19.19, 3.36]	*P* = 0.17	100%	*P* < 0.00001
Adverse events	1462	1.03[0.92, 1.15]	*P* = 0.64	40%	*P* = 0.13
Serious adverse events	1385	1.05[0.70, 1.58]	*P* = 0.80	0%	*P* = 0.69
Discontinuation	545	1.01[0.09, 10.89]	*P* = 0.99	NA	NA
Death	545	NE	NA	NA	NA
Headache	1301	0.83[0.49, 1.38]	*P* = 0.46	0%	*P* = 0.86
Injection site reactions	1421	1.43[0.93, 2.21]	*P* = 0.10	0%	*P* = 0.66
Nasopharyngitis	1385	1.09[0.78, 1.54]	*P* = 0.61	31%	*P* = 0.20
Gastroenteritis	571	1.15[0.49, 2.66]	*P* = 0.75	31%	*P* = 0.22
Nausea	652	0.67[0.28, 1.62]	*P* = 0.37	47%	*P* = 0.13
Upper respiratory tract infections	701	1.03[0.53, 1.99]	*P* = 0.93	0%	*P* = 0.37
AST or ALT>3ULN	622	1.49[0.24, 9.10]	*P* = 0.67	0%	*P* = 0.62
CK>5ULN	622	0.63[0.17, 2.29]	*P* = 0.48	28%	*P* = 0.25

WMD, weighted mean difference; RR, risk ratio; CI, confidence interval; LDL-C, low-density lipoprotein cholesterol; HDL-C, high-density lipoprotein cholesterol; TC, total cholesterol; Lp(a), lipoprotein(a); ApoA1, apolipoprotein A1; ApoB, apolipoprotein B; TG, triglycerides; AST, aspartate aminotransferase; ALT, alanine aminotransferase; CK, creatine kinase; ULN, upper limit of normal; NA, not applicable; NE, not estimable.

**Table 3:** Subgroup analyses with regard to the percentage change in plasma level of LDL-C

Subgroup	Patients, n	WMD (95% CI)	*P* value	*I*^2^,%	Heterogeneity *P* value	*P* value for subgroup differences
Adjustment for type of FH						*P* < 0.00001
HeFH	1826	−51.03%[-55.59, -46.48]	*P* < 0.00001	100%	*P* < 0.00001	
HoFH	49	−31.00%[-33.96, -28.04]	*P* < 0.00001	NA	NA	
Adjustment for type of PCSK9 antibody						*P = 0.78*
Alirocumab	1330	−49.28%[-54.95, -43.60]	*P* < 0.00001	100%	*P* < 0.00001	
Evolocumab	545	−47.21%[-60.28, -34.15]	*P* < 0.00001	99%	*P* < 0.00001	
Adjustment for duration of treatment						*P = 0.17*
≤12 weeks	622	−43.54%[-55.57, -31.51]	*P* < 0.00001	99%	*P* < 0.00001	
>12 weeks	1253	−53.02%[-59.05, -47.00]	*P* < 0.00001	100%	*P* < 0.00001	

LDL-C, low-density lipoprotein cholesterol; WMD, weighted mean difference; CI, confidence interval; FH, familial hypercholesterolemia; HeFH, heterozygous familial hypercholesterolemia; HoFH, homozygous familial hypercholesterolemia; PCSK9, proprotein convertase subtilisin/kexin type 9; NA, not applicable.

Seven trials assessed high-density lipoprotein cholesterol (HDL-C), five total cholesterol (TC), six lipoprotein (a) (Lp(a)), six apolipoprotein A1 (ApoA1), seven ApoB and six triglycerides (TG) (Table 2). HDL-C level was significantly increased with PCSK9 antibodies (mean change: 6.29%, 95% CI: 5.12 to 7.46). However, the mean changes in TC, Lp(a), ApoA1, ApoB and TG were -31.08% (95% CI: -35.20 to -26.95), -20.44% (95% CI: -25.21 to -15.66), 4.86% (95% CI: 3.77 to 5.95), -36.32% (95% CI: -40.75 to -31.90) and -7.92% (95% CI: -19.19 to 3.36), respectively. We used a random-effects model to analyze HDL-C, TC, Lp(a), ApoA1, ApoB and TG because of the significant heterogeneity (all *P* < 0.00001, *I*^2^ = 97% to 100%). The changes in lipid levels with and without PCSK9 antibodies were significant, except for a decrease in TG level (*P* = 0.17).

#### Safety outcomes

We evaluated the adverse events for the eight trials and compared the data for clinical safety outcomes (Table 2). PCSK9 antibody treatment for FH did not increase the rate of adverse events (pooled rate: 50.86 % *vs*. 48.63%; pooled relative risk [RR]: 1.03; 95% CI: 0.92 to 1.15; *P* = 0.64; heterogeneity *P* = 0.13; *I*^2^ = 40%) or serious adverse events (pooled rate: 7.14% *vs*. 6.74%; RR: 1.05; 95% CI: 0.70 to 1.58; *P* = 0.80; heterogeneity *P* = 0.69; *I*^2^ = 0%) as compared with placebo. The incidence of increased aspartate aminotransferase or alanine aminotransferase (AST or ALT) level greater than three times the upper limit of normal (ULN) did not differ with and without PCSK9 antibody (pooled rate: 0.94% *vs*. 0.51%; RR: 1.49; 95% CI: 0.24 to 9.10; *P* = 0.67; heterogeneity *P* = 0.62; *I*^2^ = 0%). The pooled incidence of increased creatine kinase (CK) level greater than five times the ULN was similar with the two treatments(pooled rate: 0.94% *vs*. 1.53%; RR: 0.63; 95% CI: 0.17 to 2.29; *P* = 0.48; heterogeneity *P* = 0.25; *I*^2^ = 28%). In addition, for other adverse events, the rates of nasopharyngitis, headache, gastroenteritis, upper respiratory tract infections and injection-site reactions were greater but not significantly with than without PCSK9 antibodies.

### Sensitivity/subgroup analyses

Sensitivity analysis was used to determine whether exclusion of any single study altered pooled RRs or weighted mean differences (WMDs). We found no heterogeneity for safety outcomes but found heterogeneity for efficacy outcomes, which was not addressed well by sensitivity analysis. Then, we performed subgroup analysis of changes in lipid and apolipoprotein levels after PCSK9 antibody treatment by different PCSK9 antibodies, types of FH and duration of treatment and found that the heterogeneity was caused in part by the different types of FH.

### Publication bias

We calculated N_fs0.05_ to estimate the publication bias for each comparison and found N_fs0.05_ values were greater than the number of studies except for incidence of serious adverse events, death, discontinuation, headache, upper respiratory tract infections and increased AST/ALT and CK included in the pooled analysis. The N_fs0.05_ value for several safety outcomes was smaller than the number of included studies, which may be consistent with “small study” bias.

## DISCUSSION

To our knowledge, this is the first pooled analysis of studies comparing the efficiency and safety of PCSK9 antibodies to no anti-PCSK9 antibodies for FH. Treatment with PCSK9 antibodies was associated with significantly reduced levels of LDL-C, TC, ApoB, and Lp(a) and elevated levels of HDL-C and ApoA1 in FH patients, with no difference in adverse events or serious adverse events with and without treatment.

In eight phase 2 and phase 3 trials that were eligible for the pooled analysis (1,879 FH patients) [25–29], the clinical adverse events with PCSK9 antibody treatment mainly concerned headache, injection-site reactions, nasopharyngitis, gastroenteritis, nausea, and upper respiratory tract infections. The total rate of adverse events or serious adverse events with treatment did not differ from the control rate. As well, laboratory analyses, including increased ALT/AST (> 3ULN) or CK (> 5ULN) levels, did not reveal a significant difference in safety issues between the two treatments. Therefore, on the strength of available data, PCSK9 antibody therapy for FH seems safe and tolerated, but more standardized trials and clinical trials are needed to further verify the safety.

We found great heterogeneity in lipid profile analyses of patients with PCSK9 antibody treatment. On sensitivity and subgroup analyses, the heterogeneity was partly caused by the different types of FH (HoFH or HeFH). Usually, patients with HeFH at least have one normal LDLR allele [3], but in HoFH patients, two LDLR alleles are abnormal [30]. Most HoFH patients are compound heterozygotes with defective LDLR-alleles [31] and others are LDLR-negative. PCSK9 antibodies might be more efficacious in reducing LDL-C level in FH patients with residual LDLR function.

FH is caused by loss-of-function mutations in the LDLR gene, leading to cell uptake of plasma LDL-C blocked by the liver and highly increased serum LDL-C level [3, 32]. Elevated LDL-C level, which is associated with atherosclerosis in affected arteries, is a major risk factor for the occurrence and development of coronary artery disease [33, 34]. Statins reduce the plasma concentration of LDL-C by increasing the hepatic expression of LDLR and removing LDL in circulation. At present, intensive statin therapy is widely indicated as first-line therapy in FH [3] to reduce serum LDL-C level and the risk of coronary artery disease [35]. However, despite high-intensity statin therapy, achieving the recommended treatment targets of LDL-C to prevent cardiovascular events is difficult in most patients with FH [3, 11, 36]. About 10% of patients are unable to tolerate high-intensity statins because of the side effects [37–39].

In recent years, combined treatment with statins and other lipid-lowering drugs has been a good therapeutic strategy to further reduce LDL-C levels for patients with FH [40]; the monoclonal antibody against PCSK9 is an innovative lipid-lowering drug. In our pooled-analysis of phase 2 and phase 3 clinical trials, treatment with PCSK9 antibodies combined with statins for FH, was effective in reducing LDL-C level, with few side effects [25–29]. In addition, statin therapy upregulates serum PCSK9 levels [15, 41, 42], and treatment with PCSK9 antibodies might strengthen statins to lower LDL-C level. So combined treatment with PCSK9 antibodies and statins may have a synergistic effect in lowering LDL-C level.

The RUTHERFORD-2 trial involved 329 HeFH patients with statins, with or without ezetimibe, randomly assigned to receive evolocumab 140 mg subcutaneously every 2 weeks or 420 mg every 4 weeks or placebo [26]. Compared with placebo, treatment with evolocumab biweekly or monthly led to 59.2% and 61.3% reduction in mean LDL-C level, respectively, after 12 weeks.

In the trial of Stein and colleagues, the efficacy and tolerability of alirocumab were evaluated in 77 patients with HeFH in the United States and Canada [27]. Alirocumab at 150 to 300 mg was found generally safe and efficacious. As well, alirocumab dose-dependently reduced LDL-C level by 28.9% to 67.9% *versus* 10.7% in the placebo group.

The TESLA Part B trial included 50 patients with HoFH who received evolocumab 420 mg or placebo every 4 weeks for 12 weeks; 49 patients actually received the study drug and completed the study [28]. Treatment with evolocumab significantly reduced LDL-C level by 30.9% as compared with placebo.

Moreover, in our analysis, other lipid levels were modified by PCSK9 antibody, including significant decreases in Lp(a), TC and ApoB levels and increase in HDL-C and ApoA1 levels. In addition, TG level was changed, although not significantly. The change in lipid profile is not conducive to the occurrence and development of atherosclerosis [43].

In patients with FH, PCSK9 antibody therapy satisfactorily regulates lipid levels, especially reducing serum level of LDL-C. Our pooled analysis revealed the good safety and tolerant profile with short-term administration of PCSK9 antibodies for FH. Results of ongoing trials of PCSK9 antibodies for FH, to evaluate the efficiency, safety and clinical outcomes with long-term treatment, are awaited.

## MATERIALS AND METHODS

This pooled analysis was conducted following the preferred reporting items of the systematic reviews and meta-analysis (PRISMA) statement. [44]

### Selection criteria

Studies were eligible for the pooled analysis if they 1) were RCTs, 2) involved human subjects with FH, and 3) compared PCSK9 antibody to no PCSK9 antibody regardless of other lipid-lowering therapy. Studies not meeting these criteria, non-clinical studies, non-RCTs and studies without complete data were excluded.

### Search sources and strategy

We performed a literature search of MEDLINE *via* PubMed, EMBASE, the Cochrane Library databases, ClinicalTrials.gov and Clinical Trial Results (www.clinicaltrialresults.org) for reports of clinical trials and RCTs published in any language up to November 30, 2015, by using the following keywords: “PCSK9” or “proprotein convertase subtilisin/kexin type 9” or “bococizumab” or “AMG 145” or “evolocumab” or ‘REGN727” or “SAR236553” or “alirocumab” and “familial hypercholesterolemia”. Reference lists of relevant trials and reviews were manually checked for additional reports.

### Data management and quality assessment

Abstracted data included first author's name, year of publication, study design, number of enrolled patients, follow-up duration, baseline characteristics of patients, drug interventions, clinical outcomes and adverse events. We recorded percentage change in lipid and apolipoprotein levels after treatment with PCSK9 antibody as the primary end point. The incidence of adverse events was a secondary end point.

Two reviewers (BL and PPH) assessed report eligibility and abstracted data independently by using a standardized report form and evaluated the quality of reports independently following the Jadad scale [45]. Any discrepancies were resolved by consensus.

### Statistical analysis

The pooled analysis involved use of REVMAN 5.3. Heterogeneity among studies was tested by the Cochran Q test and *I*^2^ test. A fixed-effects or random-effects model was applied depending on the heterogeneity results [46]: with lack of heterogeneity (*P* > 0.10 or *I*^2^ < 50%), the fixed-effects model was used, and with significant heterogeneity (*P* < 0.10 or *I*^2^ > 50%), the random-effects model was used. Two-tailed *P* < 0.05 was considered statistically significant, and RR or WMD was reported with 95% CIs. Furthermore, we performed sensitivity and subgroup analyses to lessen the influence of heterogeneity by removing an individual trial or classifying the studies based on similar features. Finally, publication bias was assessed by the fail-safe number (N_fs_): risk of publication bias was suggested if the calculated N_fs_ was less than the number of observed studies. The N_fs0.05_ was calculated as N_fs0.05_ = (∑Z/1.64)^2^-*k*, where “*k*” is the number of studies in the pooled analysis.
